# Clinical Worsening of Charcot-Marie-Tooth Disease Due to Overlapping Acute Inflammatory Polyneuropathy

**DOI:** 10.7759/cureus.47750

**Published:** 2023-10-26

**Authors:** Joana Pinto, Mariana Santos, Diana Matos, Andreia Ferreira, Ana Filipa Santos

**Affiliations:** 1 Neurology, Hospital de Braga, Braga, PRT; 2 Neuroradiology, Hospital de Braga, Braga, PRT; 3 Neurology, Alto Minho Local Health Unit, Hospital de Santa Luzia, Viana do Castelo, PRT

**Keywords:** acute inflammatory demyelinating polyneuropathy (aidp), hereditary neuropathy, neuropediatrics, neuromuscular disorders, charcot-marie-tooth disease

## Abstract

We report a case of a six-year-old male with Charcot-Marie-Tooth disease (CMT) type 1B due to* MPZ* gene mutation who experienced an acute worsening of his symptoms a few years after the diagnosis. He was not able to walk without assistance and had transitory paresthesia in his hands, 10 days after suffering from an upper respiratory and diarrheal illness. The investigation revealed elevated cerebrospinal fluid (CSF) protein levels with no pleocytosis, and sensory and motor chronic demyelinating neuropathy without active denervation findings on electrophysiological studies. The patient completely recovered following treatment with intravenous immunoglobulin. We describe the patient's history and engage in a review of the literature to find similar clinical cases. It has been proposed that *MPZ* gene mutations can change the myelin structure and result in abnormal exposure of the nervous cell components to immune cells. Hence, patients with this type of CMT would be predisposed to concurrent inflammatory forms of neuropathy.

## Introduction

Charcot-Marie-Tooth disease (CMT) is the most common inherited disease of the peripheral nervous system and it encompasses a group of genetically heterogeneous disorders. This genetic variability manifests in different patterns of inheritance (autosomal dominant, autosomal recessive, and X-linked), and there is a growing list of genes that cause or contribute to the clinical picture of these neuropathies. However, over 90% of CMT patients show a mutation in one of four following genes: peripheral myelin protein 22 (*PMP22*), mitofusin 2 (*MFN2*), myelin protein zero (*MPZ*), or gap junction protein beta 1 (*GJB1*) gene. Autosomal dominant is the most common pattern of inheritance [1,2].

CMT is further classified into electrophysiologic categories based on motor nerve conduction velocities: demyelinating (<38 m/s), axonal (>38 m/s), and intermediate (between 35 and 45 m/s). The most recent advances in molecular biology techniques have enabled the identification of more genes involved in CMT. In fact, the CMT classification comprises four main groups: CMT1, with autosomal dominant demyelinating forms; CMT2, which includes autosomal dominant axonal neuropathies; CMT3, also known as Déjérine-Sottas syndrome, which encompasses early-onset severe conditions caused by mutations in *PMP22*, *MPZ*, *PRX*, *ERG2,* and other genes and is no longer considered a separate category; CMT4, which corresponds to all autosomal recessive demyelinating types. Each category has additional subclassifications, which have been assigned letters based on the specific causal gene and phenotype [1-3].

All CMT forms share a common clinical phenotype, characterized by distal weakness, wasting and sensory loss, areflexia, and skeletal deformities with pes cavus and hammer toes. Wasting is more pronounced in the anterior compartment of the leg and gives the appearance of an “inverted champagne bottle”. A history of sprains, recurrent childhood foot fractures, and inability to walk on heels and toes are common features [1-3]. CMT is a slowly and gradually progressive disease, and it is not prone to acute exacerbations. However, there have been reports of patients who, after an initial stable course, have demonstrated accelerated worsening of their symptoms, probably related to superadded inflammation and at least partially responsive to immunomodulatory therapy [4,5].

## Case presentation

A six-year-old male was referred to our center due to frequent falls and difficulty in getting up from the floor. He had started walking late, by the age of three years, and had a family history of CMT on his father's side of the family. Neurological examination revealed mild weakness [Medical Research Council (MRC) scale: 4/5] involving distal muscles of hands and feet, diffuse absence of tendon reflexes, steppage gait, and pes cavus with hammer toes. Next-generation sequencing (NGS) of the *MPZ* gene revealed the presence of a heterozygous variant c.335T>C (p.(Ile112Thr)), the same variant that had been previously identified in his father and older brother. At the age of 11 years, he presented with a two-week history of rapid worsening of his muscle strength with an inability to walk and perform activities of daily living without assistance. He also had transient paresthesia of the hands. This deterioration had started 10 days after he had experienced an upper respiratory infection and diarrheal illness. At that time, a neurological examination had shown the proximal weakness of the lower limbs (MRC scale: 4/5), tactile and painful hypoesthesia in gloves and socks pattern, bilateral dysmetria in finger-to-nose and heel-to-chin tests, and a wide-based gait, not previously noticed. He had a mild weakness (MRC scale: 4/5) involving distal muscles in the upper limbs, but there were no cranial nerves or proprioception deficits.

Routine hematological and biochemical studies were normal. MRI of the brain and spinal cord were unremarkable. Cerebrospinal fluid (CSF) protein level was elevated (1.18 g/L) with no pleocytosis and normal glucose; antiganglioside antibodies were negative. Electrophysiological studies (Table 1) demonstrated a sensory and motor chronic demyelinating neuropathy with the absence of sensory nerve action potential of sural nerve bilaterally and motor conduction velocities of 29 m/s registered in the right median nerve; there were no observed conduction blocks, temporal dispersion, or active denervation signs. The patient was treated with intravenous immunoglobulin (2 g/kg for five days, in divided doses, once a day) and he made a full recovery to the previous state over the next weeks.

**Table 1:** Conduction velocity studies

Conduction velocity studies					
	Latency	Amplitude		Conduction velocity	FM Latency
	ms	mV	%	m/s	ms
F wave right medianus					
Wrist – abductor pollicis brevis					61.4
Motor right medianus					
Wrist – abductor pollicis brevis	4.46	10.5			
Elbow – wrist	13.5	7.4	-29.5%	29.9	
Sensory right medianus					
Digit III – wrist	3.52	16.9		35.5	
Sensory left suralis					
Middle lower leg – lateral malleolus	__	__			
Sensory right suralis					
Middle lower leg – lateral malleolus	­__	__			
Motor right tibialis					
Ankle – hallux abductor	__	__			
F wave right ulnaris					
Wrist – abductor digiti minimi					51.9
Motor right ulnaris					
Wrist – abductor digiti minimi	3.35	11.9			
Elbow – wrist	9.27	9.8	-17.6	40.5	
Sensory right ulnaris					
Digit V – wrist	3.02	17.0		36.4	

## Discussion

The* MPZ* gene is located on chromosome 1q21-q22 and encodes a major transmembrane protein whose expression is highly restricted to myelinating Schwann cells. MPZ protein is found in the compact myelin and is the most abundant structural protein of peripheral myelin sheaths. Mutations in the *MPZ* gene are associated with two main subsets of phenotypes: CMT1B (with infantile-onset symptoms and slow nerve conduction velocities) and CMT2I/2J (with adult-onset symptoms and nerve conduction velocities at the axonal range) [6,7]. The replacement of an isoleucine by a threonine found in our patient is located on the extracellular domain of MPZ protein, which is involved in homophilic interactions and is crucial for the normal compaction of myelin [6,8].

Classically, the course of CMT is gradually progressive with long periods of stability or mild decline [5]. However, some CMT patients experience a stepwise course with periods of acute worsening, which are not congruous with the natural history of the disease. Most of them present with clinical, laboratory, neurophysiological, and neuropathological features suggestive of a concomitant inflammatory neuropathy and show some response to immunomodulatory treatments. Although no data on prevalence is available, there are several case reports in the literature about patients with CMT who experience an acute clinical worsening related to an overlapping inflammatory process [4-10]. In the particular case of CMT caused by mutations in the *MPZ* gene, it has been postulated that poor myelin compaction, resulting from missense mutations, allows the circulating immune elements access to normally sequestered endoneurial components. According to this hypothesis, these patients could have genetic susceptibility to immunologic neuropathy [9]. Despite this, cases of superadded inflammation have already been described in CMT1A with *PMP22* gene duplication or CMTX with *GJB1* gene mutations, which indicates that this phenomenon is not genotype-specific. There should be a different mechanism to produce an inflammatory neuropathy associated with mutations on the *GJB1* gene that encodes a gap junction protein [5].

The first clinical sign of hereditary neuropathy in our patient was a delay in starting gait. He manifested a steady course of symptoms until the age of 11 years. Then, he had an accelerated worsening of weakness, mostly in the lower limbs, associated with paresthesia of the hands, almost two weeks after suffering from an infectious disease. This clinical presentation associated with albuminocytologic dissociation on CSF was suggestive of an acute inflammatory neuropathy. Electrophysiological studies were conducted one month after the onset of the symptoms and two weeks after the end of the treatment; this temporal gap probably explains the lack of evidence for classical signs of acquired demyelination such as conduction blocks or temporal dispersion signs. Treatment with a single cycle of intravenous immunoglobulin completely resolved the acute symptoms and our patient returned to his basal state.

Although the pathophysiological mechanism of CMT remains unknown, there is evidence regarding a subgroup of CMT patients with superimposed inflammatory neuropathy. This can partly explain the phenotypic variability seen between individuals with the same genotype. Although the long-term beneficial effect of the immunomodulatory treatments is uncertain, the recognition of an immunological component creates new therapeutic opportunities for some CMT patients beyond rehabilitation, auxiliary walking devices, orthopedic surgery, and genetic counseling [5,10].

## Conclusions

In our patient, despite the diagnosis of CMT1B, the acute worsening of weakness, accompanied by positive sensory symptoms and ataxia, elevated CSF protein levels, and previous infectious disease suggested an inflammatory neuropathy. Recognition of a superimposed inflammatory process was of great clinical importance since the subsequent immunomodulatory treatment contributed to improving the outcome in our patient.
